# Supplementary material for: Relative Validity and Reproducibility of a Semi-Quantitative Web-Based Food Frequency Questionnaire for Swiss Adults
Source: Nutrients. 2025 Apr 30;17(9):1555. doi: 10.3390/nu17091555 (PMC12073886; doi:10.3390/nu17091555)
Supplement: Supplementary file 1 [file nutrients-17-01555-s001.zip › nutrients-3573100-supplementary.pdf]

Supplementary material: Relative validity and reproducibility of a semi-quantitative web-based food frequency questionnaire for Swiss adults

Supplementary methods: Data management

All study data were self-reported and were collected using the following data collection methods: 1) Redcap® software[1, 2], was used to manage and interact with study participants, and to collect all non-dietary intake related data via integrated surveys; dietary intake data were collected using 2) the Swiss eFFQ web application[3] and 3) paper-based 4-d Food records. For electronic data collection methods 1 and 2, study participants received instructions and an invitation link to the surveys via e-mail, whereas for method 3, analogous documents were sent to participants by mail. At the end of the study, data from the different sources were exported as Excel or csv files, imported into R software (version 4.3.1 for Windows, R Foundation for Statistical Computing, Vienna, Austria), and merged by an automatically generated and anonymous participant ID.

Supplementary material: Relative validity and reproducibility of a semi-quantitative web-based food frequency questionnaire for Swiss adults

Supplementary figures and tables

Table S1: Original wording and corresponding labels of the usability questionnaire completed by study participants at T_0.

Assigned English label	Wording of the usability questionnaire (French)	Wording of the usability questionnaire (German)
Strongly disagree | Disagree | Somewhat disagree | Neither agree nor disagree | Somewhat agree | Agree | Strongly agree	**Vous trouverez ci-dessous des informations spécifiques au questionnaire alimentaire en ligne. Veuillez préciser ce qui vous semble le plus juste :** Fortement en désaccord | En désaccord | Plutôt en désaccord | Ni en désaccord, ni d'accord | Plutôt d'accord | D'accord | Fortement d'accord	**Im Folgenden finden Sie spezifische Aussagen über den Ernährungsfragebogen. Bitte geben Sie an, was Ihrer Meinung nach am ehesten zutrifft:** Trifft überhaupt nicht zu | Trifft nicht zu | Trifft eher nicht zu | Teils teils | Trifft eher zu | Trifft zu | Trifft voll und ganz zu
Intuitive to use	J'ai trouvé l'utilisation du questionnaire alimentaire intuitive.	Ich fand die Bedienung des Ernährungsfragebogens intuitiv.
Visual design appealing	J'ai trouvé la présentation visuelle du questionnaire alimentaire attrayante.	Ich fand die visuelle Gestaltung des Ernährungsfragebogens ansprechend.
User interface structured	J'ai trouvé l'interface utilisateur du questionnaire alimentaire structurée.	Ich fand die Benutzeroberfläche des Ernährungsfragebogens strukturiert.
Instruction clear and easy to understand	J'ai trouvé les instructions pour remplir le questionnaire alimentaire claires.	Ich fand die Anweisungen zum Ausfüllen des Ernährungsfragebogens klar verständlich.
Questions formulated clearly	J'ai trouvé que les questions étaient formulées de manière compréhensible.	Ich fand die Fragen verständlich formuliert.
Food pictures helpful	J'ai trouvé les images des aliments utiles.	Ich fand die Lebensmittelbilder hilfreich.
Frequency options suitable	J'ai trouvé les choix de fréquence de consommation (par ex. 2-3 fois par mois) appropriés.	Ich fand die Auswahlmöglichkeiten zur Häufigkeit passend (z.B. monatlich: 2-3 mal).
FFQ easy to answer	J'ai trouvé facile de répondre au questionnaire alimentaire.	Ich fand den Ernährungsfragebogen einfach zu beantworten.
Willing to fill out again	Je serais partante de le remplir à nouveau à l'avenir.	Ich wäre dazu bereit, den Ernährungsfragebogen auch in Zukunft nochmals auszufüllen.
Recommend to others	Je recommanderais le questionnaire alimentaire à mon entourage.	Ich würde den Ernährungsfragebogen Freunden und Kollegen weiterempfehlen.
Why not recommend to others?	Pourquoi ne recommanderiez-vous pas le questionnaire alimentaire à votre entourage ?	Warum würden Sie den Ernährungsfragebogen eher nicht an Freunde und Kollegen weiterempfehlen?
Further comments/suggestions	Si vous avez d'autres remarques ou suggestions concernant le questionnaire alimentaire, vous pouvez les indiquer ici :	Falls Sie weitere Anmerkungen oder Anregungen zum Ernährungsfragebogen haben, können Sie diese in den folgenden Zeilen angeben:

Supplementary material: Relative validity and reproducibility of a semi-quantitative web-based food frequency questionnaire for Swiss adults

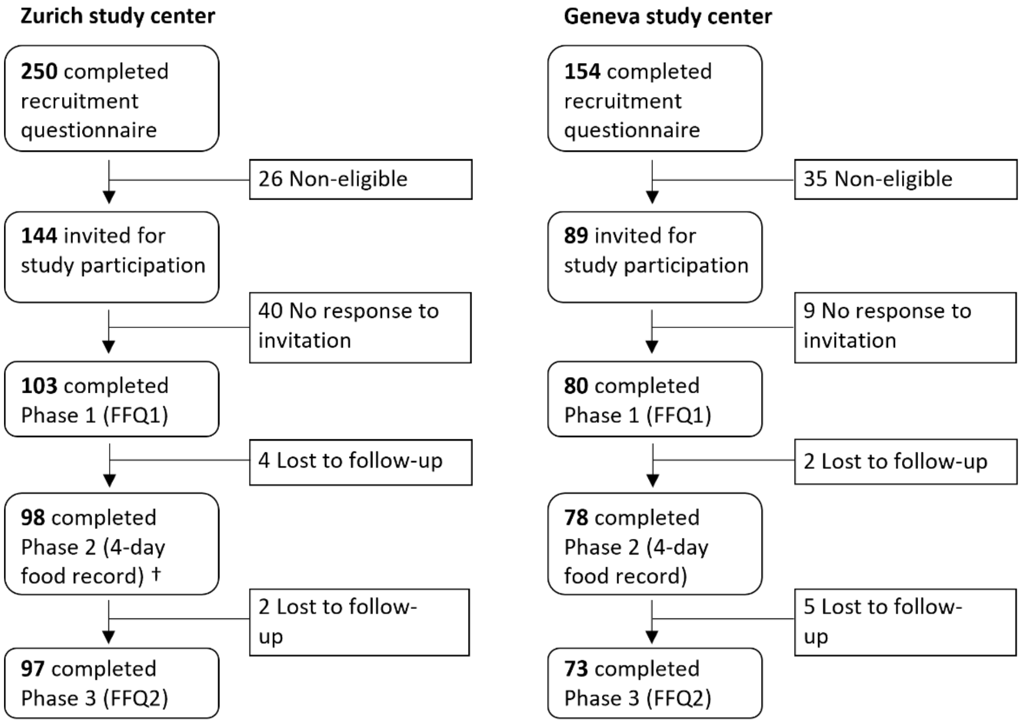

Figure S1: Flow chart of study participants in the Swiss eFFQ validation study by study center (recruitment period: Jan–Apr 2023). Participants who completed phase 1 were officially enrolled in the study (n=183), but only participants who took part in at least 2 completed phases were analyzed (n=177).
Two study participants (one per study center) withdrew consent to further participate in the study and were excluded.
† One study participant (in Zurich) completed phases 1 and 3 but skipped phase 2.
FFQ1, first food frequency questionnaire collected at T_0 (Phase 1); FFQ2, second food frequency questionnaire collected at T_2 (Phase 3).

Supplementary material: Relative validity and reproducibility of a semi-quantitative web-based food frequency questionnaire for Swiss adults

Table S2: Comparison of estimated daily micronutrient intakes of the first Swiss eFFQ with the 4-d food records (relative validity, n=176) and with the second Swiss eFFQ (reproducibility, n=170).

	Relative validity (FFQ1 vs. mean of 4-d FR)						Reproducibility (FFQ1 vs. FFQ2)						
Dietary intake variable	FFQ1 Median (IQR)	4-d FR Median (IQR)	% Group-level bias†	% SQ / SOAQ‡	% EQ‡	K_w §	FFQ2 Median (IQR)	% Group-level bias†	SCC ‖	LCC	ICC	% SQ / SOAQ‡	% EQ ‡
Cholesterol (mg/d)	225.9 (179.1, 292.9)	260 (192.4, 367.7)	-17.1***	33.5/72.7	5.1	0.25	223.2 (164.8, 275.7)	7.7***	0.67***	0.69	0.70	48.2/90.0	3.5
Total sugar (g/d)	71.8 (54.4, 93.9)	77.1 (60.4, 106.2)	-11.9***	38.1/83.0	5.1	0.35	62.0 (49.0, 88.2)	9.1***	0.71***	0.75	0.76	52.4/91.2	1.2
Water (g/d)	2794.1 (2323.8, 3193.5)	2735.2 (2263.2, 3201.0)	-3.0	33.5/76.1	4.0	0.27	2750.4 (2299.0, 3229.4)	1.4	0.72***	0.71	0.72	47.6/88.8	0.6
Calcium (mg/d)	729.3 (593.6, 950.6)	911.9 (689.1, 1179.3)	-16.9***	35.8/75.0	7.4	0.20	668.8 (531.6, 882.9)	8.8***	0.74***	0.61	0.63	55.9/94.1	1.8
Iodine (µg/d)	66.9 (54.1, 81.6)	80.6 (62.0, 106.2)	-20.0***	37.5/69.3	10.2	0.16	64.5 (50.6, 81.9)	3.5*	0.69***	0.67	0.67	47.6/88.8	1.2
Iron (mg/d)	7.8 (6.1, 9.8)	10.3 (8.5, 12.8)	-27.0***	37.5/73.9	8.0	0.21	7.3 (5.8, 9.2)	3.9*	0.68***	0.69	0.70	49.4/90.0	1.2
Magnesium (mg/d)	257.8 (212.1, 320.8)	335.2 (264.0, 428.5)	-23.0***	31.8/69.3	5.1	0.24	249.4 (203.6, 311.2)	4.3*	0.69***	0.68	0.69	48.8/88.8	1.2
Phosphorus (mg/d)	1068.3 (906.3, 1321.9)	1337.6 (1137.4, 1633.5)	-19.4***	38.6/69.9	9.1	0.16	1031.2 (822.0, 1295.5)	5.5***	0.69***	0.64	0.65	51.8/88.2	1.2
Potassium (mg/d)	2336.4 (1955.3, 2782.8)	2876.9 (2437.8, 3505.4)	-20.3***	34.1/75.6	7.4	0.27	2238.6 (1796.2, 2793.7)	3.7*	0.67***	0.63	0.63	47.1/87.1	2.4
Zinc (mg/d)	8.2 (6.7, 9.9)	9.9 (8.1, 12.6)	-22.6***	34.1/76.1	12.5	0.20	7.6 (6.0, 9.7)	4.8**	0.70***	0.67	0.67	52.9/91.8	2.4
Salt (g/d)	4.2 (3.1, 5.2)	5.7 (4.2, 7.4)	-28.4***	35.8/76.7	7.4	0.22	3.9 (3.0, 5.0)	5.2***	0.79***	0.80	0.80	52.9/91.8	0.0
Sodium (mg/d)	1782.2 (1294.0, 2284.9)	2370.4 (1796.0, 3095.2)	-26.8***	34.7/77.8	7.4	0.26	1644.5 (1285.9, 2166.8)	5.8***	0.78***	0.79	0.80	55.9/92.3	0.0

ATE, Alpha-tocopherol equivalents; EQ, extreme (opposite) quartile; FFQ1, first food frequency questionnaire collected at T_0 (Phase 1); FFQ2, second food frequency questionnaire collected at T_2 (Phase 3); ICC, Intraclass correlation coefficients; IQR, interquartile range; K_w, weighted Cohen's kappa; LCC, Lin's concordance correlation coefficients; RE, retinol equivalents (RE = 1 x retinol (µg) + 1/6 x beta carotene equivalent (µg-BCE)); SCC, Spearman's correlation coefficients; SOAQ, same or adjacent quartile; SQ, same quartile; 4-d FR, 4-day Food record.
*p < 0.05, **p < 0.01, ***p < 0.001.
† Relative validity: Group-level bias = (mean intake FFQ1) / (mean intake 4-d FR) * 100 – 100); Reproducibility: Group-level bias = (mean intake FFQ1) / (mean intake FFQ2) * 100 – 100); Wilcoxon signed-rank test assessed with FFQ1 and 4-d FR, and FFQ1 and FFQ2, respectively.
‡ Cross-classification analysis results are expressed as percentage of participants classified in the same/same or adjacent, and extreme quartile of nutrient distribution by FFQ1 and the 4-d FR (relative validity) and FFQ1 and FFQ2 (reproducibility). Values are missing if distributions did not allow the creation of unique quartiles.
§ Calculation of K_w is based on tertiles. Values are missing if distributions did not allow the creation of unique tertiles.
‖ Spearman's p-value approximation was used to assess statistical significance of SCCs.

Supplementary material: Relative validity and reproducibility of a semi-quantitative web-based food frequency questionnaire for Swiss adults

Table S2 (continued): Comparison of estimated daily micronutrient intakes of the first Swiss eFFQ with the 4-d food records (relative validity, n=176) and with the second Swiss eFFQ (reproducibility, n=170).

	Relative validity (FFQ1 vs. mean of 4-d FR)						Reproducibility (FFQ1 vs. FFQ2)						
Dietary intake variable	FFQ1 Median (IQR)	4-d FR Median (IQR)	% Group-level bias†	% SQ / SOAQ‡	% EQ‡	K_w §	FFQ2 Median (IQR)	% Group-level bias†	SCC ‖	LCC	ICC	% SQ / SOAQ‡	% EQ ‡
Retinol (µg/d)	301.1 (237.0, 476.1)	358.3 (271.4, 521.6)	-22.6**	36.9/75.6	4.6	0.27	283.8 (203.1, 370.3)	19.0***	0.66***	0.45	0.46	51.8/89.4	2.9
Vitamin A activity (µg-RE/d)	747.7 (560.5, 1038.9)	1160.1 (780.5, 1720.1)	-36.1***	30.7/71.0	6.8	0.18	682.9 (523.8, 889.7)	12.8***	0.65***	0.55	0.57	53.5/85.9	1.8
Vitamin B1 (mg/d)	0.9 (0.7, 1.1)	1.1 (0.9, 1.3)	-22.1***	36.9/73.9	10.8	0.16	0.8 (0.7, 1.1)	4.9**	0.68***	0.70	0.71	58.2/87.1	2.9
Vitamin B2 (mg/d)	1.1 (0.8, 1.4)	1.3 (1.1, 1.6)	-19.6***	33.0/71.6	6.2	0.26	1.0 (0.8, 1.3)	4.4**	0.71***	0.66	0.67	56.5/89.4	2.4
Vitamin B6 (mg/d)	1.2 (1.0, 1.5)	1.5 (1.3, 1.9)	-20.0***	31.8/74.4	9.7	0.13	1.2 (1.0, 1.5)	2.7*	0.67***	0.65	0.65	50.6/87.7	2.4
Folate (µg/d)	215.9 (179.0, 263.2)	271.5 (211.4, 343.4)	-21.8***	36.9/81.2	5.1	0.30	203.7 (159.2, 248.7)	7.0***	0.70***	0.67	0.68	52.4/91.2	0.6
Vitamin B12 (µg/d)	3.4 (2.5, 5.6)	3.4 (2.4, 5.2)	2.6	37.5/74.4	5.7	0.29	3.2 (2.4, 5.0)	6.8**	0.74***	0.65	0.65	52.9/91.2	1.2
Vitamin C (mg/d)	97.8 (70.1, 126.8)	121 (86.9, 169.1)	-21.4***	37.5/79.0	1.7	0.27	76.2 (60.7, 103.8)	23.2***	0.57***	0.48	0.53	45.3/82.9	1.2
Vitamin D (µg/d)	2.5 (2.0, 3.2)	2.7 (1.8, 4.2)	-16.8**	34.7/75.0	6.8	0.26	2.4 (1.7, 3.2)	4.7**	0.70***	0.61	0.62	51.2/87.1	0.6
Vitamin E activity (mg-ATE/d)	11.9 (9.4, 15.2)	12 (9.6, 14.9)	-1.3	34.7/70.4	6.8	0.16	11.6 (9.1, 15.1)	5.1*	0.69***	0.61	0.62	47.1/90.0	2.4

ATE, Alpha-tocopherol equivalents; EQ, extreme (opposite) quartile; FFQ1, first food frequency questionnaire collected at T_0 (Phase 1); FFQ2, second food frequency questionnaire collected at T_2 (Phase 3); ICC, Intraclass correlation coefficients; IQR, interquartile range; K_w, weighted Cohen's kappa; LCC, Lin's concordance correlation coefficients; RE, retinol equivalents (RE = 1 x retinol (µg) + 1/6 x beta carotene equivalent (µg-BCE)); SCC, Spearman's correlation coefficients; SOAQ, same or adjacent quartile; SQ, same quartile; 4-d FR, 4-day Food record.
*p < 0.05, **p < 0.01, ***p < 0.001.
† Relative validity: Group-level bias = (mean intake FFQ1) / (mean intake 4-d FR) * 100 – 100); Reproducibility: Group-level bias = (mean intake FFQ1) / (mean intake FFQ2) * 100 – 100); Wilcoxon signed-rank test assessed with FFQ1 and 4-d FR, and FFQ1 and FFQ2, respectively.
‡ Cross-classification analysis results are expressed as percentage of participants classified in the same/same or adjacent, and extreme quartile of nutrient distribution by FFQ1 and the 4-d FR (relative validity) and FFQ1 and FFQ2 (reproducibility). Values are missing if distributions did not allow the creation of unique quartiles.
§ Calculation of K_w is based on tertiles. Values are missing if distributions did not allow the creation of unique tertiles.
|| Spearman's p-value approximation was used to assess statistical significance of SCCs.

Supplementary material: Relative validity and reproducibility of a semi-quantitative web-based food frequency questionnaire for Swiss adults

Table S3: Association and limits of agreement of estimated daily nutrient intakes between the first Swiss eFFQ and 4-d food records and variance ratios based on 4-d FRs (n=176).

Dietary intake variable	SCC (crude)†‡	SCC (de-attenuated) ‡	SCC (energy adj. de-attenuated) ‡	Variance ratio §	% LoA ‖
Energy (kcal/d)	0.28***	0.30	.	0.72	96.0
Carbohydrates (g/d)	0.40***	0.43	0.49	0.70	94.9
Carbohydrates (E%/d)	0.47***	0.53	0.51	0.96	93.8
Dietary fiber (g/d)	0.48***	0.52	0.66	0.74	94.9
Dietary fiber (E%/d)	0.61***	0.66	0.66	0.67	94.9
Protein (g/d)	0.28***	0.31	0.42	0.92	96.0
Protein (E%/d)	0.40***	0.44	0.42	0.98	92.6
Fat (g/d)	0.22**	0.24	0.46	0.91	92.6
Fat (E%/d)	0.40***	0.45	0.47	1.16	94.9
Fatty acids monounsaturated (g/d)	0.11	0.13	0.32	1.07	94.3
Fatty acids polyunsaturated (g/d)	0.33***	0.36	0.48	0.96	93.2
Fatty acids saturated (g/d)	0.41***	0.46	0.56	0.83	93.2
Alcohol (g/d)	0.72***	0.77	0.72	0.58	95.5
Alcohol (E%/d)	0.70***	0.75	0.72	0.54	94.9
Cholesterol (mg/d)	0.39***	0.46	0.35	1.41	96.0
Total sugar (g/d)	0.49***	0.52	0.48	0.54	93.8
Water (g/d)	0.37***	0.38	0.33	0.39	95.5
Calcium (mg/d)	0.34***	0.39	0.46	1.20	95.5
Iodine (µg/d)	0.24**	0.28	0.37	1.42	95.5
Iron (mg/d)	0.31***	0.34	0.42	0.89	94.9
Magnesium (mg/d)	0.33***	0.36	0.68	0.72	94.9
Phosphorus (mg/d)	0.24**	0.26	0.37	0.83	93.2
Potassium (mg/d)	0.32***	0.35	0.55	0.82	93.8
Zinc (mg/d)	0.23**	0.25	0.19	0.71	97.2
Salt (g/d)	0.38***	0.43	0.36	0.95	95.5
Sodium (mg/d)	0.40***	0.45	0.36	0.95	95.5
Retinol (µg/d)	0.45***	0.59	0.46	2.99	98.9
Vitamin A activity (µg-RE/d)	0.24**	0.31	0.28	2.63	97.7
Vitamin B1 (mg/d)	0.27***	0.30	0.41	0.97	93.8
Vitamin B2 (mg/d)	0.37***	0.40	0.47	0.74	95.5
Vitamin B6 (mg/d)	0.24**	0.27	0.37	1.18	94.9
Folate (µg/d)	0.44***	0.48	0.64	0.74	94.9
Vitamin B12 (µg/d)	0.37***	0.45	0.45	2.07	92.6
Vitamin C (mg/d)	0.46***	0.50	0.51	0.85	94.9
Vitamin D (µg/d)	0.35***	0.44	0.48	2.17	94.3
Vitamin E activity (mg-ATE/d)	0.27***	0.30	0.37	0.90	96.0

Adj., adjusted; ATE, Alpha-tocopherol equivalents; E%, percentage of total energy; LoA, limits of agreement; RE, retinol equivalents (RE = 1 x retinol (µg) + 1/6 x beta carotene equivalent (µg-BCE)); SCC, Spearman's correlation coefficients; 4-d FR, 4-day Food record.

† Spearman's p-value approximation was used to assess statistical significance of crude SCCs (*p < 0.05, **p < 0.01, ***p < 0.001).

‡ SCCs are crude, de-attenuated and de-attenuated residual energy-adjusted SCCs (according to the residual energy adjustment method).

§ Ratios of within- to between-person variation ($\lambda = S_w^2 / S_b^2$) were calculated from 4-d FRs using one-way ANOVA.

‖ LoA is based on Bland-Altman analysis and expresses the proportion of participants whose difference between FFQ1 and 4-d FR intake was within 1.96 ± SD of the mean population difference.

Supplementary material: Relative validity and reproducibility of a semi-quantitative web-based food frequency questionnaire for Swiss adults

Table S4: Association and limits of agreement of estimated daily food group intakes between the first Swiss eFFQ and 4-d food records and variance ratios based on 4-d FRs (n=176).

Dietary intake variable (g/d)	SCC (crude)†‡	SCC (de-attenuated) ‡	Variance ratio §	% LoA ‖
Bread and bread products	0.45***	0.50	0.96	96.0
Grains and grain products, rice	0.44***	0.52	1.58	92.6
Potatoes and potato products	0.30***	0.38	2.50	93.2
Bakery products, cakes and pastry	0.45***	0.52	1.26	94.3
Sweets, sugar, dessert and ice cream	0.50***	0.56	1.06	94.3
Meat, processed meat and sausage	0.60***	0.69	1.21	94.9
Tofu and other vegetarian meat replacements	0.49***	0.56	1.35	95.5
Milk, dairy products and cheese	0.52***	0.56	0.53	96.6
Vegetarian dairy product replacements	0.58***	0.61	0.45	91.5
Fish and seafood	0.43***	0.53	2.12	93.8
Eggs	0.33***	0.39	1.74	96.0
Vegetables	0.39***	0.43	0.95	94.9
Legumes	0.46***	0.56	1.88	95.5
Fruits and fruit products	0.61***	0.67	0.74	94.3
Nuts and seeds	0.50***	0.55	0.79	90.9
Fats, oils and cream	0.08	0.10	1.22	96.6
Savory sauces	0.04	0.04	1.21	96.0
Mixed dishes and soups	0.16*	0.20	1.85	93.2
Salty snacks	0.25***	0.32	2.25	95.5
Non-alcoholic beverages	0.39***	0.40	0.35	92.6
Alcoholic beverages	0.71***	0.78	0.79	94.9

LoA, limits of agreement; SCC, Spearman's correlation coefficients; 4-d FR, 4-day Food record.
† Spearman's p-value approximation was used to assess statistical significance of crude SCCs (*p < 0.05, **p < 0.01, ***p < 0.001).
‡ SCCs are crude and de-attenuated SCCs.
§ Ratios of within- to between-person variation ($\lambda = S_w^2 / S_b^2$) were calculated from 4-d FRs using one-way ANOVA.
‖ LoA is based on Bland-Altman analysis and expresses the proportion of participants whose difference between FFQ1 and 4-d FR intake was within 1.96 ± SD of the mean population difference.

Supplementary material: Relative validity and reproducibility of a semi-quantitative web-based food frequency questionnaire for Swiss adults

References

1. Harris PA, Taylor R, Minor BL *et al.* (2019) The REDCap consortium: Building an international community of software platform partners. *J Biomed Inform*. **95**.
2. Harris PA, Taylor R, Thielke R *et al.* (2009) Research electronic data capture (REDCap)--a metadata-driven methodology and workflow process for providing translational research informatics support. *J Biomed Inform*. **42**, 377-81.
3. Pannen ST, Gassmann R, Vorburger R *et al.* (2023) Development of a Multilingual Web-Based Food Frequency Questionnaire for Adults in Switzerland. *Nutrients*. **15**.
